# Proteomic comparison of selective breeding and growth hormone transgenesis in fish: Unique pathways to enhanced growth

**DOI:** 10.1016/j.jprot.2018.08.013

**Published:** 2019-02-10

**Authors:** Dwight R. Causey, Jin-Hyoung Kim, David A. Stead, Samuel A.M. Martin, Robert H. Devlin, Daniel J. Macqueen

**Affiliations:** a School of Biological Sciences, University of Aberdeen, Aberdeen, UK; b Fisheries and Oceans Canada, West Vancouver, British Columbia V7V 1N6, Canada; c Korea Polar Research Institute (KOPRI), Yeonsu-gu, Incheon 21990, Republic of Korea; d Aberdeen Proteomics, University of Aberdeen, Rowett Institute, Aberdeen, UK

**Keywords:** Coho salmon, Fish growth, Growth Hormone Transgenesis, Selective Breeding, Label-free High Throughput Proteomics

## Abstract

In fish used for food production and scientific research, fast growth can be achieved via selective breeding or induced instantaneously via growth hormone (GH) transgenesis (GHT). The proteomic basis for these distinct routes towards a similar higher phenotype remains uncharacterized, as are associated implications for health parameters. We addressed this knowledge gap using skeletal muscle proteomics in coho salmon (*Oncorhynchus kisutch*), hypothesising that i) selective breeding and GHT are underpinned by both parallel and unique changes in growth systems, and ii) rapidly-growing fish strains have lowered scope to allocate resources towards immune function. Quantitative profiling of GHT and growth-selected strains was done in comparison to wild-type after injection with PBS (control) or Poly I:C (to mimic infection). We identified remodelling of the muscle proteome in each growth-enhanced strain that was strikingly non-overlapping. GHT was characterized by focal upregulation of systems driving protein synthesis, while the growth-selected fish presented a larger and more diverse set of changes, consistent with complex alterations to many metabolic and cellular pathways. Poly I:C had little detectable effect on the muscle proteome. This study demonstrates that distinct proteome profiles can explain outwardly similar enhanced growth phenotypes, improving our understanding of growth mechanisms in anthropogenic animal strains.

**Significance:**

This work provides the first proteomic insights into mechanisms underpinning different anthropogenic routes to rapid growth in salmon. High-throughput proteomic profiling was used to reveal changes supporting enhanced growth, comparing skeletal muscle of growth hormone transgenic (GHT) and selectively-bred salmon strains with their wild-type counterparts. Contrasting past mRNA-level comparisons of the same fish strains, our data reveals a surprisingly substantial proteomic divergence between the GHT and selectively bred strains. The findings demonstrate that many unique molecular mechanisms underlie growth-enhanced phenotypes in different types of fish strain used for food production and scientific research.

## Highlights

• Mechanistic basis for rapid growth poorly understood in fish.
• Comparative proteomic profiling done in fish strains showing highly enhanced growth.
• Distinct basis for enhanced growth comparing transgenic and domesticated fish strains.
• Highly distinct proteome profiles may explain outwardly similar growth phenotypes.
• Study enhances understanding of how rapid growth is achieved in anthropogenic animal strains.

## Graphical abstract

**Figure:**
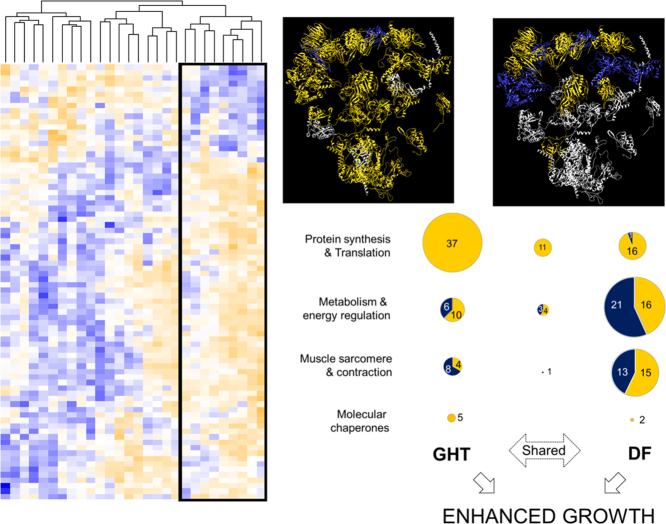

## 1 Introduction

In farmed salmonid fishes, selective breeding has been ongoing for decades leading to large increases in growth [1,2]. As an alternative to selective-breeding, highly elevated growth rate can be achieved by transgenesis within a wild-type genetic background [3] using constructs overexpressing growth hormone (GH). Stable GH transgenic (GHT) salmon strains are approved in North America as a food product (e.g. [4]), so understanding how growth is achieved by GHT in comparison to selective-breeding approaches has regulatory, risk assessment, and social implications [5,6]. Comparisons of GHT and growth-selected fish also provides insights into the potential mechanisms supporting rapid growth in anthropogenic animal strains.

An important question concerns whether rapid growth is achieved by parallel or unique molecular pathways comparing GHT and growth-selected domesticated fish. Most work has focused on GH-dependent pathways governed by insulin-like growth factors (IGFs). Pituitary secreted GH enters circulation and binds GH receptors on target tissues, activating JAK/STAT pathways [7], with diverse physiological effects. GH signalling stimulates hepatic production of IGF-I [8], which enters circulation and binds a specific receptor (IGF1R) on target cells, activating anabolic signalling [9]. IGF hormones (IGF-I and IGF-II) also influence growth in a paracrine/autocrine fashion [10,11]. Salmonids can be strongly growth stimulated by exogenous GH treatment [12], though this hormone has a greater effect in wild-type strains compared to growth-selected domesticated fishes [13], suggesting that high GH signalling was selected during domestication, indicating substantial parallels for the basis of rapid growth compared to GHT.

Growth-selected domesticated and GHT fish show parallel changes in transcriptional regulation of GH and IGF system genes, and similarities in the modulation of genes that differ in expression from wild-type [14,15]. GHT strains have elevated plasma GH and IGF-I compared to growth-selected domesticated strains, and both strains show elevated levels of these hormones compared to wild-type [2,14,16,17]. There is also more limited evidence for non-parallel molecular changes in GHT and growth-selected domesticated strains [2,14,15,17]. Growth is a polygenic trait and selective breeding offers scope to alter genetic pathways across a genome. Consequently, while GHT should only impact GH-dependent phenotypes, selective breeding can theoretically modify growth associated functions encoded anywhere in the genome. Here our primary objective was to explore these ideas systematically using proteomics, testing the hypothesis that both parallel and non-parallel molecular changes underpin enhanced growth achieved by GHT and selective breeding.

Another important question concerns how rapid growth affects other physiological functions important to fish health/production. Trade-offs exist between growth and immune function in fish (e.g. [[18], [19], [20]]) and require mechanisms to reallocate resources away from growth investment towards immune function during disease challenge; thought to involve cross-talk between the GH/IGF systems and innate immune factors [18,21]. In animal strains with enhanced growth rate, the balance of investment into competing physiological systems is heavily shifted, with potential costs on immune function reported in GHT salmon [19,22]. A secondary aim of this study was to test the hypothesis that rapidly-growing fish show disrupted immune function and altered cross-talk between growth and immune function at the proteomic level.

As a focal point, we studied the most important target tissue for growth and energy storage in fishes: skeletal muscle, where growth is centrally controlled by GH and IGF signalling pathways [23,24]. In salmonids, muscle accounts for 50–60% of mass and is used to store amino acids and lipids that can be reallocated to other systems during catabolic states. Muscle function is also regulated by cross-talk with the immune system [25], an interaction that is disrupted by GHT in salmonids [19]. Our study hypotheses were tested using high-throughput label-free proteomics [26,27] to compare the muscle proteome of fast-growing GHT versus growth-selected domesticated fish strains relative to the wild-type. We reveal substantial divergence among-strains that contrasts to a surprising extent from past mRNA-level studies, indicating that distinct anthropogenic routes to fast growth in fish are supported by several unique mechanisms.

## 2 Materials and methods

### 2.1 Experimental Design and statistical rationale

Coho salmon were sampled as part of an experiment reported in full elsewhere [19], performed at Fisheries and Oceans Canada (DFO), West Vancouver, British Columbia, Canada. The study was carried out under permit #12–017 from the DFO's Pacific Regional Animal Care Committee. The animal groups included a wild-type (WT) and a GHT strain (M77), where the transgene was inserted and subsequently maintained in the same WT genetic background [19]. While not described in our previous publication, an additional fish group was included in the same experiment, namely a domesticated aquaculture strain (hereafter: DF) selected for enhanced growth over seven generations, originally derived from the Kitimat River (British Columbia). DF were size-matched to WT and GHT (*n* = 60 DF fish were used in the experiment: mean ± s.d.: 77.9 ± 0.5 g) and all fish studied were immature, unsexed and fed to satiation. Size-matching required sampling at different ages due to differences in growth rate (WT: aged 19 months; DF: aged 10 months; GHT: aged 6 months), and all fish were sampled on the same date. Details of how the fish were treated during the experiment, including acclimation to a common garden set-up, allocation into replicated tanks, immune/control injections, killing methods and tissue sampling, was done as described in [19]. Skeletal muscle samples were flash-frozen on dry ice and stored at -70 °C until analysis.

Thirty skeletal muscle samples from the above experimental design were used in the proteomic analyses reported in this study (two were eventually dropped as outliers; see Section 2.4). Samples came from animals either 30 h post Poly I:C injection (200 μg per 100 g fish weight) to mimic a viral infection (*n* = 5 biological replicates per WT, GHT and DF) or 30 h post PBS injection (*n* = 5 biological replicates per WT, GHT and DF) to provide an unstimulated control. Technical replicates were not performed on these thirty samples because we anticipated large variation among experimental groups that would be sufficiently captured through the biological replication. The biological replicates used for proteomics were a random subset of sampled fish, as described in [19]. It was not possible to use a formal test of statistical power to inform the appropriate number of biological replicates due to a lack of pilot data on the study system. As reported within the Results section, the selected design provided sufficient power to detect differential abundance of individual proteins among experimental groups while controlling for type-I error (described in Section 2.4.).

### 2.2 Sample preparation

Muscle samples (approx. 100 mg) were thawed on ice, weighed and added to 50 μl lysis buffer (0.5 M pH 6.8 Tris-HCl, 0.2 M EDTA, 8 M Urea, 0.5 M DTT, 10% *v*/v Glycerol, 10% v/v NP40, pH 3–10 ampholytes). This solution was ground using a micropestle, before a further 50 μl lysis buffer was added and the tissue was sonicated (Sonic Dismembrator, Fisher Scientific) on ice to make a ratio of 2 mg tissue per μl lysis buffer. The resulting solution was centrifuged at 13,000*g* for 5 min, before an aliquot was separated on a 10% acrylamide 1-D gel and stained with Coomassie blue to ensure the staining intensity was matched across samples. The supernatant was then stored at -80 °C until further analysis. After thawing on ice, 50 μl of supernatant was combined with 50 μl molecular grade water. Proteins were precipitated using the ReadyPrep 2-D clean up procedure (Bio-Rad Laboratories), following the manufacturer's instructions. The resulting pellet was dissolved in 100 μl of 3–10 pH Reswell buffer (Urea, Thiourea, CHAPS, DTT, MilliQ water, and IPG buffer). 10 μl of the resulting solution was combined with 5 μl 3× dissociation buffer (0.5 M pH 6.8Tris-HCl, 25% SDS, 2-mercaptoethanol, Glycerol) and incubated for 5 min at 100 °C for denaturation. 3 μl of the extract was run a short distance into a 10% acrylamide 1-D gel, which was stained with colloidal Coomassie Blue G250 (Fischer Scientific) and the proteins excised and used for in-gel tryptic (Promega, sequencing grade) digestion (Digilab ProGest robot). We also included quality control samples consisting of a standard solution of BSA in each batch of tryptic digestions to confirm the completeness of the digestion process. Peptide solutions were dried by centrifugal evaporation (Savant SpeedVac Plus), dissolved in 20 μl 0.1% formic acid, and spun at 14,000 *g* for 5 min prior to liquid chromatography – mass spectrometry (LC-MS).

### 2.3 LC-MS

The LC-MS system used was an UltiMate 3000 RSLCnano (Dionex/Thermo Scientific) coupled to a Q Exactive Plus quadrupole-equipped Orbitrap MS (Thermo Scientific). 4 μl of tryptic peptide solution was injected for analysis per sample. Peptides were concentrated on a μ-precolumn (C18 PepMap; 300 μm i.d. × 5 mm) in a water/acetonitrile/formic acid (98:2:0.1) loading solvent at a flow rate of 10 μl/min. After 5 min, the μ-precolumn was switched to the analytical flow path, where peptides were separated at a flow rate of 0.3 μl/min on a C18 PepMap RSLC column (75 μm i.d. x 50 cm) with a particle size of 2 μm, fitted to an Easy-Spray nano ESI source. Two solvents were formed, solvent A was made from water/formic acid (1000:1) and solvent B from water/acetonitrile/formic acid (20:80:0.1). An increasing proportion of solvent B was used to separate peptides along a gradient: 3–10% from 5 to 25 min; 10–45% from 25 to 185 min; 45–90% from 185 to 190 min; 90% from 190 to 205 min; 90–3% from 205 to 210 min, followed by re-equilibration of the column (3% Solvent B, 30 min). LC lasted 240 min; mass spectra were acquired using a “Top 10” data-dependent method starting at 5 min and lasting 200 min. The electrospray voltage was 1.9 kV, capillary temperature 270 °C and S-lens RF level 60. Full MS scans were conducted between 375 and 1750 *m/z* at resolution 70,000 (*m/z* 200), automatic gain control 3E + 6 and maximum injection time 50 ms. Following each survey scan the 10 most intense ions of charge state 2–5 were sequentially selected (isolation window 1.6 *m/z*) and fragmented in the higher-energy collisional dissociation (HCD) cell at a normalized collision energy of 26%. MS/MS scans were conducted at resolution 17,500, automatic gain control 5E + 4 and maximum injection time 100 ms. Additional data-dependent settings were: peptide match preferred, exclude isotopes turned on, and a dynamic exclusion of 40 s.

### 2.4 Data and statistical analysis

Q Exactive raw data were analysed using MaxQuant v1.5.3.30 with Andromeda as the peptide search engine [28] and the label-free quantification (LFQ) method [29]. Most standard recommended settings were used [30], but to ensure maximal data discovery, the ‘Fast LFQ’ option was not applied. The trypsin digestion option was selected, along with a maximum of two missed cleavages, allowing oxidation of methionine and acetylation at the N-terminus end of the protein as variable modifications, and carbamidomethylation of cysteine as a fixed modification. The mass tolerance for first search precursor ions was set to 20 ppm followed by 4.5 ppm for the main search. A 1% false-discovery rate (FDR) was employed for peptide and protein identifications. The ‘Match between runs’ option was used to transfer identifications to other LC-MS runs and increase identifications across samples. Unmodified counterpart peptides were not discarded. Peptides and proteins were identified against a database of 57,592 RefSeq proteins predicted from the coho salmon genome annotation (NCBI accession; GCA_002021735.1, currently unpublished). Any coho salmon proteins identified in our study were confirmed against annotated RefSeq proteins from the ‘gold-standard’ Atlantic salmon (*Salmo salar*) reference ICSASG_v2 genome [31] (NCBI accession: GCA_000233375.4) using BLASTp [32]. The MaxQuant-generated ‘proteingroups.txt’ file was filtered for contaminants, reverse identifications, and proteins only identified by site.

Statistical tests and graphical functions were performed in R-studio v.1.0.136 (Rstudio, Boston, MA) interfacing with R v.3.3.2 (“Sincere Pumpkin Patch”), using the log-transformed imputed LFQ data. Only protein groups that had LFQ values in at least *n* = 3 samples per each of the six experimental groups (WT-PBS, WT-Poly I:C, GHT-PBS, GHT-Poly I:C, DF-PBS, DF-Poly I:C) were retained for analyses reported hereafter. The remaining LFQ values were log-2 transformed, and imputed to increase quantitative comparisons and decrease the effect of highly abundant proteins [33]. Imputation was completed using missForest [34], a non-parametric, random forest approach. The protein-level probabilistic imputation used has been shown to outperform peptide-level imputation [35] and our data showed high reproducibility across samples both before and after imputation (see Fig. S1; Section 3.1). To determine statistical differences among fish strains (WT vs. GHT vs. DF: fixed factors) and treatments (Poly I:C vs. PBS/control: fixed factors), we utilized a linear model approach in the ‘limma’ package, with subsequent empirical Bayes smoothing of the standard errors [36]. After determining there was no immune-related statistical differences (see RESULTS), pairwise comparisons were conducted between each strain (GHT vs. WT, DF vs. WT, and DF vs. GHT), equivalent to a post-hoc Tukey's test following ANOVA. A global false discovery rate (FDR) adjustment was applied to correct for multiple comparisons. The ‘gplots’ and ‘seriation’ packages were used to produce heat maps [37,38] comparing *Z*-score normalized LFQ values of significantly different protein abundances identified from the linear model (FDR-adjusted *P* < 0.05). Hierarchical clustering was done using optimal leaf ordering, which aims to minimize Hamiltonian path length, and reveals more biological structure than heuristic methods [39]. Further multivariate analyses were performed using the ‘vegan’ package [40]. Non-metric multidimensional scaling (nMDS) was used to visualize multivariate differences between strains and immune treatments. Permutational ANOVA [41] (PERMANOVA, 9999 permutations) was used to determine proteome-wide changes between individual fish across strains (DF vs. GHT vs. WT), immune treatment (PBS vs. Poly I:C), and the interaction of strain by treatment. The multivariate homogeneity of group dispersion (variance) was assessed to determine potential effects on multivariate statistical outcomes [42]. This revealed a dispersion effect on strain, which may influence the PERMANOVA. The dispersion and location of data was checked in an ordination plot, and it was determined that the location effect was more prominent than any multivariate data dispersion. Two samples (one WT and GHT; both PBS treatments) were outliers from other fish in the same strains based on their proteomic profiles at the univariate and multivariate levels, and removed from the study.

The complete MaxQuant output and log-transformed imputed LFQ data used in the statistical analysis are provided within Table S1 and S2, respectively. The mass spectrometry proteomics data for the 28 samples reported in the study have been deposited to the ProteomeXchange Consortium via the PRIDE [43] partner repository with the dataset identifier PXD009537.

### 2.5 Mapping changes in ribosome protein abundance to the 80S ribosome structure

Changes in the abundance of ribosomal proteins among GHT and DF compared to WT were visualized on a 3D structural model of a vertebrate 80S ribosome. The 5 Å resolution cryo-electron microscopy-derived 80S ribosome structure of human (*Homo sapiens*) (accession: 4V6X) [44] was downloaded from the RCSB Protein Data Bank and rendered/edited according to our findings in UCSF Chimera v.1.10.2 [45]. Coho salmon ribosomal proteins (present within the final dataset) orthologous to those within the human 80S structure were determined by BLASTp.

## 3 Results

### 3.1 Overview of proteomic data

Among a larger set of protein identifications (Table S1), 320 proteins were retained for analysis following quality-control steps and stringent filtering of the data to include only proteins identified in at least *n* = 3 individuals per experimental group. On average, the proteins retained for analysis were identified in 27.1 (SD: 1.66 samples) out of the 28 samples used in the statistical analysis reported below, so represent highly-reproducible proteins in the coho salmon skeletal muscle proteome. As shown in Fig. S1, we also observed a high level of repeatability among protein abundances across the 28 samples, both considering log2-transformed LFQ values (Pearson's *R* = 0.82–0.99 across samples) and imputed values for the same data (Pearson's *R* = 0.84–0.99 across samples).

### 3.2 Multivariate analyses

**Fig. 1:**
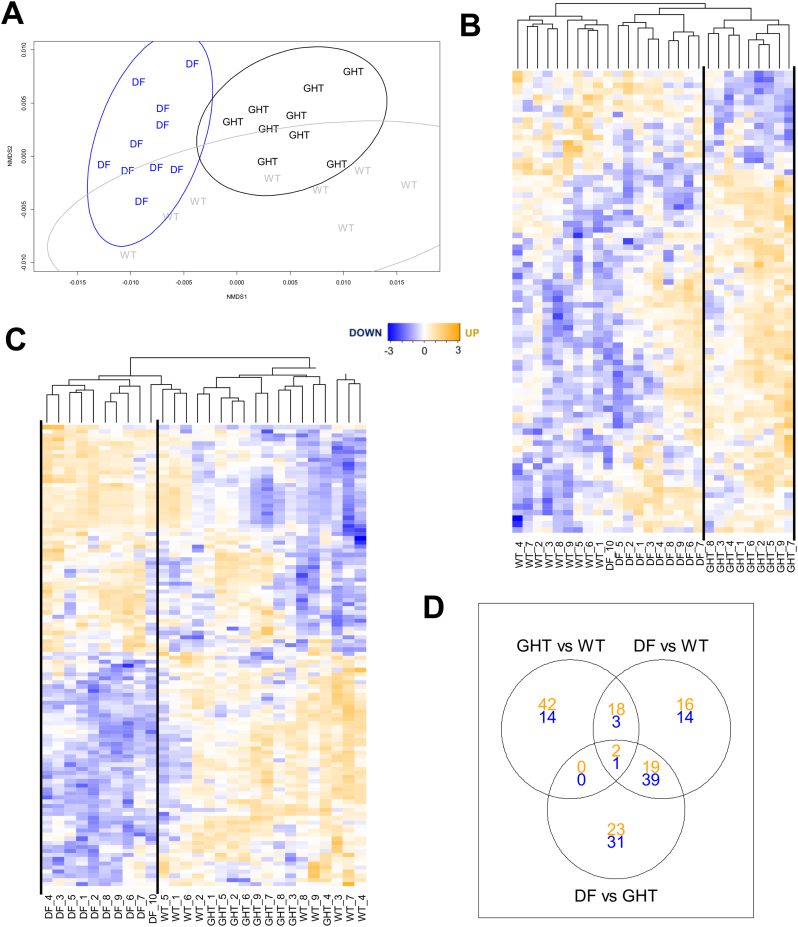
(A). Proteome-wide nonmetric multidimensional scaling analysis of wild-type (WT), growth-hormone transgenic (GHT), and domesticated fish (DF) possessing distinct growth rates. Each label represents an individual fish and ellipses represent 95% confidence intervals around strain groupings. Also shown is a hierarchical clustering and heatmap analysis demonstrating global differences in proteins showing significantly different abundances comparing (B) GHT and WT to (C) DF and WT. In each heatmap, all fish individuals from the three different strains are included for comparison. Rows represent normalized *Z*-scores of label-free quantification (LFQ) values from MaxQuant. Separate heatmaps for the three pairwise strain comparisons (i.e. A and B above, as well as GHT vs. DF) including protein identifications are provided in Figs. S3–5. (D) Venn diagram showing the number of significantly different protein abundances common to each pairwise strain comparison (orange: upregulated; blue: downregulated). (For interpretation of the references to colour in this figure legend, the reader is referred to the web version of this article.)

Using the 320-protein dataset in an nMDS analysis revealed a proteome-wide separation by fish strain (DF vs. GHT vs. WT) (Fig. 1A) but not by immune treatment (PBS vs. Poly I:C) (Fig. S2). Consistently, PERMANOVA indicated a highly significant strain effect (*pseudo*-F_2,27_ = 6.63, *P* = 0.0001), but non-significant effects for treatment (*pseudo*-F_1,27_ = 2.10, *P* = 0.054) and a strain:treatment interaction (*pseudo*-F_2,27_ = 1.62, *P* = 0.083).

### 3.3 Univariate analyses

Consistent with the multivariate analyses, a linear model considering all proteins separately revealed that the abundance of 191 proteins (59.7% of total) was significantly affected (FDR-adjusted *P* < 0.05) by fish strain (Table S3). Conversely, just 5 proteins (<1%) were significantly altered by Poly I:C across strains (Table S4). Among the proteins with a significant overall strain effect, 80 had differential abundance between GHT and WT (FDR-adjusted *P* < 0.05; Table S3). In a hierarchical clustering analysis of these proteins across strains, GHT samples grouped within a larger cluster including all but one DF individual (Fig. 1B). Among 62 proteins upregulated in GHT compared to WT, more than half are ribosomal proteins, while others are involved in translation (e.g. elongation factor [EF] 1-gamma, eukaryotic translation initiation [eIF] factor 5A), energy metabolism (e.g. fructose-1,6-bisphosphatase 2; phosphoglycerate mutase 2), sarcomere organization (e.g. troponin-I/-C, myozenin-1-like) or represent molecular chaperones (HSP60, 70, 90, and HSPA8) (Fig. S3, Table S3). The sarcomeric proteins nebulin and myosin heavy chain feature among the 18 downregulated proteins in GHT compared to WT, along with proteins involved in connective tissue (collagen alpha-3 and prolargin-like), calcium channel function (voltage-dependent L-type calcium channel subunit b) and lipid metabolism (trifunctional enzyme subunit alpha, mitochondrial) (Fig. S3, Table S3).

Among the proteins showing a strain effect, 112 (58.6% of total) had significantly different abundances between DF and WT (Table S3). Hierarchical clustering analysis of these proteins revealed a major DF cluster within a larger grouping containing three WT individuals, separate from a cluster containing GHT and WT individuals (Fig. 1C). In contrast to the GHT vs. WT comparison, a similar number of proteins were upregulated (55 proteins) and downregulated (57 proteins) (Fig. 1C; Table S3). WT fish that grouped with DF had similar abundance profiles for many downregulated proteins, and a smaller set of upregulated proteins, but showed the WT pattern for other proteins (Fig. 1C). Proteins with diverse functions were altered in the DF strain compared to WT, including ribosomal and sarcomeric proteins, proteins involved in muscle contraction (e.g. parvalbumin), kinases (e.g. nucleoside diphosphate kinase A, adenylate kinase, glycogen phosphorylase), molecular chaperones (e.g. HSP70 and 90-alpha), translation (e.g. EF1-gamma & EF1-alpha, eIF4A-I & eIF4H), calcium regulation (e.g. parvalbumin, voltage-dependent L-type calcium channel subunit beta-1), signalling molecules (e.g. RACK1, 14–3-3 protein beta/alpha-2 and epsilon-like) and a range of metabolic enzymes (e.g. betaine-homocysteine methyltransferase, glucose-6-phosphate isomerase, aldolase, glyceraldehyde-3-phosphate dehydrogenase) (Fig. S4; Table S3).

Among the proteins showing a strain effect, 115 (60.2%) had significantly different abundances between DF and GHT (Table S3). In the hierarchical clustering analysis of these proteins, DF fish formed a cluster within a grouping that contained the same three WT individuals mentioned above (Fig. S5). The remaining GHT and WT fish formed a separate cluster where individuals did not cluster by strain (Fig. S5). Among the proteins differing between DF and GHT, the majority (71) were downregulated in DF, including ribosomal proteins, metabolic enzymes, translation factors, molecular chaperones and kinases (Fig. S5; Table S3). The 44 upregulated proteins included sarcomeric proteins, translation factors and metabolic enzymes (Fig. S5; Table S3).

The 5 proteins significantly affected by Poly I:C included three downregulated (gamma-enolase, creatine kinase, myosin heavy chain) and two upregulated (troponin-I and ribosomal protein S13) proteins (Table S4).

### 3.4 Limited proteome changes common to GHT and DF

An obvious difference in the number of proteins with altered abundance comparing GHT vs. WT and DF vs. WT led us to question the extent of shared and non-overlapping differences across strains. 21 proteins showing altered abundance (18 upregulated, 3 downregulated) were shared by DF and GHT when separately compared to WT, versus 58 (19 upregulated, 39 downregulated) for both GHT and WT compared to DF (Fig. 1D). The 18 commonly upregulated proteins in DF and GHT compared to WT included 10 ribosomal proteins. 19 commonly upregulated proteins in GHT and WT compared to DF included EFs, kinases, and sarcomeric proteins, while the 39 commonly downregulated proteins included metabolic enzymes and a distinct set of kinases and sarcomere proteins. A marked subset of differential protein abundances was restricted to each of the pairwise strain comparisons (Fig. 1D).

To supplement the above broad-scale comparisons, below we describe, in greater depth, changes in classes of proteins particularly important to growth and muscle function.

### 3.5 Protein synthesis and breakdown

**Fig. 2:**
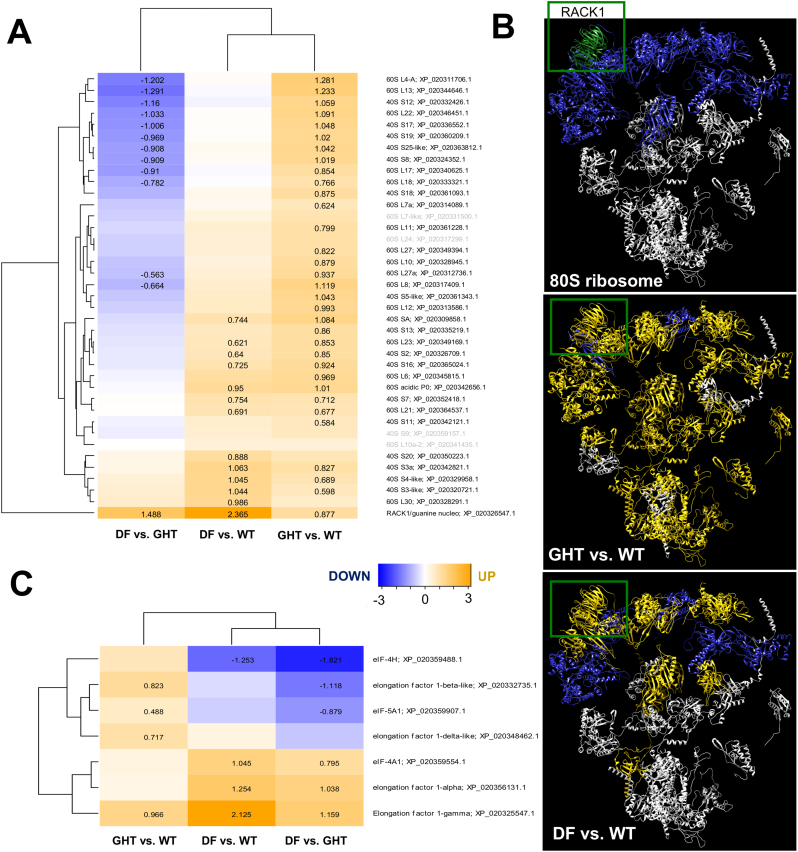
Differences in the abundance of proteins from systems driving protein synthesis in GHT and DF compared to WT. (A) Hierarchical clustering of all identified ribosomal proteins. Proteins with titles in black showed significant differential abundance across the salmon strains. Log2 fold-changes are given for proteins with significant differential abundance between the shown pairwise strain comparisons. Accession numbers and protein names are from the *O. kisutch* and *S. salar* NCBI RefSeq databases, respectively. (B) The functional relevance of changes in ribosomal protein abundance was explored by mapping the data onto a 3D structure of the human 80S ribosome [44], where any ribosomal proteins lacking orthologous salmon proteins in our dataset were removed, along with ribosomal RNA. The top panel shows all proteins orthologous to proteins in the human 80S structure, with 40S (small subunit) and 60S (large subunit) proteins shaded blue and white respectively. RACK1 is shown in green. In the lower panels, proteins showing upregulation in GHT or DF compared to WT are shaded gold. (C) Hierarchical clustering of eukaryotic translation elongation and initiation factors with details as for part B. (For interpretation of the references to colour in this figure legend, the reader is referred to the web version of this article.)

Proteins driving the increased protein synthesis necessary to support rapid growth are represented within our data and associated with the ribosomal machinery. Remarkably, 85% (33/39) of all detected cytoplasmic ribosomal proteins were significantly upregulated in GHT compared to WT, equally representing the small/40S (16 proteins) and large/60S (16 proteins) subunits (Fig. 2A), as visualized on a 3D structural model of the 80S ribosome (Fig. 2B). A smaller set of 13 ribosomal proteins, 11 overlapping with GHT, were upregulated in DF compared to WT, more biased to the 40S (8 proteins) than 60S (4 proteins) subunit (Fig. 2A, C). A substantial fraction of ribosomal proteins upregulated in GHT compared to WT were significantly downregulated in DF relative to GHT (Fig. 2A), highlighting substantial differences in ribosome biogenesis in the two fast growing strains. Several eIFs and EFs showed alterations in GHT and DF compared to WT, which were largely non-overlapping (Fig. 2C). EF1-gamma showed increased abundance in both GHT and DF, while EF1-alpha was upregulated specifically in DF vs. both GHT and WT, and EF1-delta and EF1-beta specifically in GHT vs. WT (Fig. 2C). While eIF-4A1 was upregulated in DF compared to both GHT and WT, eIF-4H was downregulated in the same comparison, while eIF-5A1 increased in GHT compared to both DF and WT (Fig. 2C). RACK1, a component of the 80S ribosome (Fig. 2B), showed higher abundance in both GHT and DF compared to WT, but was particularly upregulated (approx. 4.4-fold) in DF vs. WT, and also higher in DF than GHT (Fig. 2A, Table S3).

On the catabolic side of protein turnover, we identified a group of proteins involved in muscle protein breakdown, including subunits of the 26S proteasome, along with a limited representation of the calpain system (i.e. calpastatin) (Fig. S6A). Contrasting the substantial upregulation of proteins driving protein synthesis, our data indicates that muscle protein breakdown pathways are largely unaltered in both rapid growing coho salmon strains, with only one 26S proteasome subunit showing significant upregulation in DF vs. WT and no proteins altered significantly between GHT and WT (Fig. S6A).

### 3.6 Molecular chaperones

The molecular chaperone group of heat-shock proteins (HSP) showed a partly overlapping increase in abundance in DF and GHT compared to WT (Fig. S6B). HSP 90-alpha and HSP70 were elevated in both DF and GHT vs. WT (Fig. S6B). HSP60 and HSPA8 (Hsc71) were upregulated specifically in GHT compared to WT, with HSPA8 being significantly higher in GHT than DF (Fig. S6B). The only other identified molecular chaperone (78 kDa glucose-regulated protein) showed no significant changes across strains.

### 3.7 Energy metabolism

**Fig. 3:**
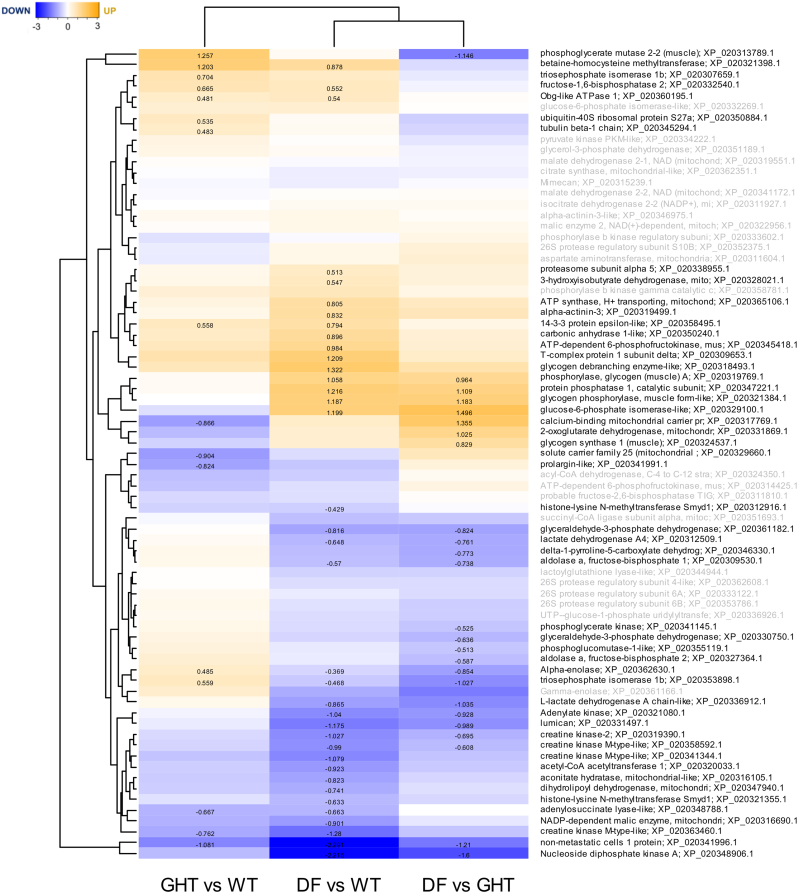
Differences in the abundance of proteins from metabolism and energy regulating systems in two growth-enhanced salmon strains, GHT and DF, compared to WT. Other details are as given in the Fig. 2 legend.

Among 79 proteins with identified functions in metabolism and energy regulation that had significantly different abundances across fish strains, 37 (47%) differed significantly between DF and WT, and 16 (20%) between GHT and WT (Fig. 3). A modest proportion (7/16) of the proteins showing differential abundances between GHT and WT were altered in the same direction comparing DF and WT, but this represents a small number of all proteins showing differential abundance between DF and WT. A larger group of 16 proteins showed common changes in abundance comparing DF to both GHT and WT, and there were also a substantial number of unique differences comparing DF and GHT (Fig. 3). Among the significantly upregulated proteins in DF compared to GHT and WT was glycogen phosphorylase, glucose-6-phosphate isomerase, glycogen synthase, and glycogen debranching enzyme (Fig. 3). A serine/threonine-protein phosphatase (PPP) beta catalytic subunit was downregulated in DF compared to both GHT and WT (Fig. 3). Adenylate kinase and two nucleoside diphosphate kinase A variants (one annotated as ‘non-metastatic cells 1 protein’) showed lower abundance in DF compared to both GHT and WF (Fig. 3). Betaine-homocysteine methyltransferase and fructose-1,6-bisphosphatase 2, along with two additional metabolic proteins, were increased in both DF and GHT compared to WT (Fig. 3).

### 3.8 Sarcomeric organization and muscle contraction

The abundance of muscle sarcomere proteins and proteins with functions in muscle contraction showed marked changes in DF compared to WT and GHT (Fig. S7). A set of proteins fundamental to sarcomere organization, including titins, myosin heavy chains, and myomesins showed upregulation in DF compared to both GHT and WT, several of which were downregulated in GHT compared to WT (Fig. S7). It is striking to note that all proteins upregulated in DF vs. both GHT and WT represent structural proteins. This contrasts with a distinct cluster of strongly downregulated proteins in DF compared to both GHT and WT, dominated by proteins involved in muscle contraction regulation, many of which are calcium-regulated, including troponin-I and -C, myosin light/regulatory light chains and parvalbumin (Fig. S7).

## 4 Discussion

**Fig. 4:**
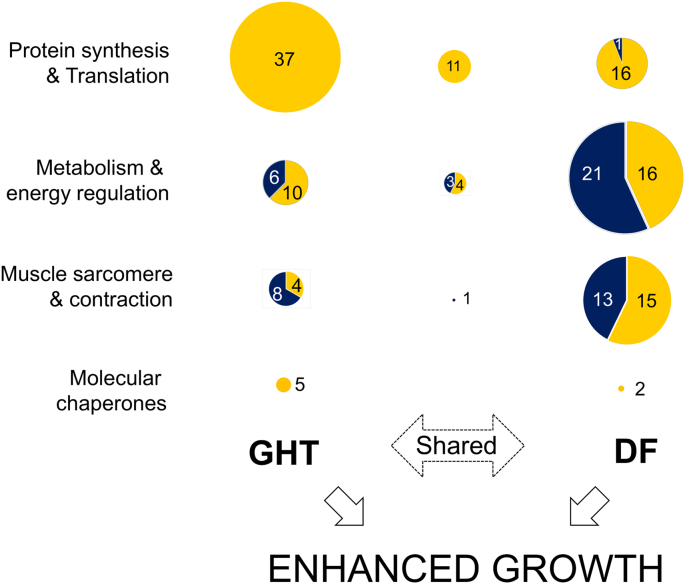
The distinct proteomic basis for enhanced growth in skeletal muscle of GHT and DF coho salmon strains. Circles are scaled in diameter to the number of proteins that showed significantly different abundances comparing GHT vs. WT and DF vs. WT. Circles are shaded to highlight the number of proteins (values indicated) showing increased abundance (yellow) and reduced abundance (dark blue). Using the same scheme, we highlight the number of proteins showing common significant differences in abundance comparing GHT and DF to WT. (For interpretation of the references to colour in this figure legend, the reader is referred to the web version of this article.)

This study demonstrates the value of label-free high throughput proteomics for dissecting the mechanistic basis of complex traits, using fish strains with different genetic characteristics as a model. A key finding was that while the skeletal muscle proteomes of two growth-enhanced coho salmon strains were remodelled from wild-type (WT), there were differences depending on the underlying basis for enhanced growth (summarized in Fig. 4). While a GH transgenic (GHT) strain showed a particularly focal upregulation of systems driving protein synthesis with relatively limited impacts on other systems, a domesticated strain (DF) subjected to generations of selective-breeding for enhanced growth showed a more diverse and predominantly non-overlapping set of proteomic changes, more consistent with a polygenic basis for enhanced growth. Interestingly, the WT genetic background remained highly visible in the GHT strain, as many proteomic changes distinguished DF from WT and GHT in the same way. In such respects, our primary study hypothesis was supported, yet the extent of non-parallel molecular changes comparing GHT and DF (to WT) has not been observed in past mRNA-level studies [2,14,15,17]. Several factors may explain this difference. First, mRNA does not always correlate with protein abundance [46]. Second, many genes targeted or detected at the mRNA-level in past studies of the same fish strains, including genes from the GH and IGF systems [[14], [15], [16]], were not detected in our analysis. This can be explained by a relatively small fraction of proteins, particularly structural and metabolic proteins, being preferentially detected in the MS and MS/MS scans due to their high abundance, limiting power to detect low-abundance proteins such as hormones. However, given that protein levels are a more direct reflection of phenotype than mRNA, the strain-specific proteomic differences reported here are relevant for our understanding of growth mechanisms, and perhaps warrant caution when using comparisons at the mRNA level to explain phenotypic differences among strains.

Our data provided little evidence that Poly I:C markedly alters the skeletal muscle proteome, opposing our second study hypothesis. This was surprising, as our recent study with the same WT and GHT muscle samples demonstrated that Poly I:C induced antiviral and growth gene expression responses that differed among strains [19]. This apparent contradiction may be explained by our inability to detect changes in low-abundance proteins regulating immune function, e.g. cytokines. However, PERMANOVA revealed a near significant proteome-wide effect of immune treatment as well as treatment by strain interaction. It is therefore possible that the stringency of statistical analyses applied contributed to a reduced power to detect immune-responsive proteins. Future high throughput LC/MS proteomics studies of muscle may benefit from targeted MS (i.e. data independent analysis), longer run times, cellular fractionation, or other approaches to increase power to detect less abundant proteins. Additional work will be required to fully dissect the proteomic basis for cross-talk between growth and immune function in fishes.

An important study finding was the striking upregulation of systems driving protein synthesis in growth-enhanced salmon strains, which was particularly exaggerated in GHT. This constituted an increased abundance of many ribosomal proteins from both the small (40S) and large (60S) subunits that are essential for protein synthesis [47,48]. As circulating IGF-I is increased in both GHT and DF compared to WT [14,16,17], some of the observed parallel increases in ribosome protein abundance may potentially be driven by the PI3K-Akt-mTOR pathway [49], which is activated by IGF-I and regulates the transcription and translation of many ribosomal proteins [50]. However, our findings also suggest that GH per se, rather than its impact on increased endocrine IGF-I secretion, is responsible for upregulation of the much larger set of ribosomal proteins specific to GHT. Similarly, while the rapid growth phenotype of both GHT and DF is characterized by upregulation of eIFs and EFs essential for protein synthesis [47], the same proteins showed largely non-overlapping changes among the two growth-enhanced strains. For example, EF1-alpha was upregulated in DF specifically, which may be explained by DF-specific changes in growth factors beyond GH or IGF—I, such as epidermal growth factor [51]. Additional EFs were upregulated in GHT but not DF (e.g. EF1-delta and EF1-beta), suggesting their regulation depends on GH signalling specifically, rather than the impact of GH on IGF—I. It also seems important that RACK1 was particularly strongly upregulated in DF. This scaffolding protein represents a key ribosomal component that is vital for the efficient translation of short mRNAs with housekeeping functions, including ribosomal proteins [52]. Beyond its central role in translation, RACK1 interacts with a diverse set of proteins to modulate a broad range of molecular pathways and functions spanning various cell compartments (reviewed in [53]). Hence, the strong RACK1 upregulation in DF muscle is very likely to mediate phenotypic changes that extend beyond enhanced protein synthesis. Given the lack of observed regulation in protease systems in both GHT and DF compared to WT, our data suggests that enhanced growth is not being accomplished by a reduction in the rate of muscle protein breakdown in either strain.

Our data also suggests greater remodelling in energy metabolism pathways in skeletal muscle of DF than in GHT, characterized by unique changes in abundance of carbohydrate-processing proteins, including upregulation of several enzymes involved in glycolysis, glycogenolysis, gluconeogenesis, and the pentose phosphate pathway (e.g. glucose-6-phosphate isomerase, glycogen phosphorylases, glycogen debranching enzyme and protein phosphatase 1) [[54], [55], [56]]). However, the situation is clearly complex, as DF showed downregulation of several proteins involved in glucose breakdown and rapid energy generation, including L-lactate dehydrogenase B chain, creatine kinase-2, and creatine kinase M-type, among others. While it's well established that carbohydrates play a less significant dietary role in carnivorous fishes such as coho salmon than amino acids and lipids, they do have a role in supporting muscle energy demands, and play crucial roles in intermediary metabolism (reviewed in [57,58]). It is also known that salmonids show strain variation in the efficiency of carbohydrate metabolism [58], implying the presence of genetic variation that could be selected during the domestication process. Therefore, we speculate that DF-specific remodelling of muscle energy metabolism resulted from selection for increased efficiency of energy generation or intermediate metabolism during the domestication process to support growth via pathways that use glucose to generate ATP and other key metabolites (e.g. NADPH and ribose 5-phosphate) necessary to support the costs of protein turnover, along with anabolic processes linked to energy storage (e.g. lipogenesis). During the domestication process, salmon have had the chance to adapt to commercial diets with a radically distinct composition from natural diets, including in terms of carbohydrate, amino acid and lipid content/profile, with scope to impact carbohydrate metabolism directly, but also through diverse mechanisms that allow cross-talk with systems regulating lipid and amino acid metabolism [57,58]. Such adaptation would not be expected in GHT fish, which have been back-crossed with WT fish to avoid selection effects from domestication [3]. Nonetheless, past work showed that GHT salmon have enhanced carbohydrate metabolism at the enzyme, nutritional, and physiological levels [59,60], whereas at the mRNA level, changes indicative of enhanced carbohydrate metabolism were observed among GHT and DF in liver [14,61] and to some extent skeletal muscle [62]. Therefore, the comparative lack of remodelling observed in GHT muscle indicative of altered carbohydrate metabolism could reflect a tissue-specific effect in DF that is independent of GH and its downstream impacts on growth. These findings warrant further proteomic studies incorporating additional tissues, notably liver as a centre for carbohydrate storage and metabolism.

Another notable difference between DF and GHT compared to WT was the downregulation of adenylate kinase (AK) in DF. This enzyme acts to increase ADP and AMP in the absence of ATP [63], which activates the key energy sensor AMP-activated protein kinase (AMPK) [64]. AK deficiency significantly reduces AMPK phosphorylation, activity, and efficacy in skeletal muscle [65,66]. The reduction of AK in DF muscle predicts downstream repression of AMPK activation, which is consistent with the high-energy state required to achieve rapid growth. However, given that many proteins involved in glycolysis and carbohydrate metabolism are altered in DF, changes in AK could also relate to this proteins AMP-mediated impacts on glycolytic enzymes that are AMPK-independent [65]. The lack of AK downregulation in GHT implies that alternative pathways may exist to regulate AMPK signalling.

An interesting metabolic change shared between DF and GHT is upregulation of betaine-homocysteine methyltransferase, indicating a dietary influx of betaine, which leads to downstream formation of phospholipids important for cellular membrane generation [67]. Increased dietary betaine is associated with improved growth after smoltification in salmonids, owing to a reduction in osmoregulatory stress [68], which is notable as fast-growing salmon strains show accelerated smoltification and osmoregulatory ability [3,69].

Unique alterations in skeletal muscle function provide another level distinguishing GHT and DF from each other and WT. The DF strain experienced an increase in many proteins essential to sarcomere organization and structure, while several proteins involved with muscle contraction were downregulated. Conversely, fewer proteins in the same functional classes were altered in GHT, and almost none showed parallel changes to those observed between DF and WT. The role played by such strain differences remains unclear, but is likely to underlie differences in muscle composition and contractile properties. Finally, our results suggest remodelling of molecular chaperone protein abundance supports the rapid growth of DF and GHT, involving some parallel, but largely non-parallel changes in abundance of heat-shock protein (HSP) family members. In a general sense, upregulation of HSPs occurs under stressful cellular conditions, including in cancer cells in a growth context (reviewed in [70]), acting to ensure proper protein folding. HSP70 and HSP90-alpha were commonly upregulated in DF and GHT. Both proteins are essential to rapid cell growth in their chaperone function [70], while HSP90-alpha promotes cellular growth through regulation of transcription machinery acting downstream of hormonal pathways [71,72]. Additionally, HSPA8/Hsc70 and HSP60, which have diverse functions in ensuring proper assembly and folding of proteins in multiple cellular compartments [[73], [74], [75]], were increased specifically in GHT, suggesting increased intrinsic cellular stress compared to DF, perhaps related to higher rates of protein synthesis driven by increased translation.

## 5 Conclusions

GHT and selective breeding for increased growth rate are underpinned by a surprisingly non-overlapping remodelling of the skeletal muscle proteome. These findings have implications for commercial fish production, especially in terms of the design of feeds matched to the metabolic requirements of distinct growth-enhanced fish strains. It will be interesting to determine whether independent episodes of fish domestication and selection for enhanced growth, including in other animal species, are accompanied by similar changes to our observations. Given the importance of the GH and IGF systems as drivers of increased growth rate, future proteomics studies might focus on phosphorylation-driven changes in downstream signal transduction pathways, which represent key intermediates to our findings that remain largely unexplored in fishes. Finally, our data have implications for environmental risk assessments of anthropogenic fish strains that may enter natural environments [76]. While GHT and domesticated fish strains may outwardly appear to possess similar phenotypic characteristics (e.g. growth rate, enhanced feeding motivation, among others), our data demonstrate that the underlying basis for such differences can be markedly different, and thus caution against making regulatory decisions generic to all ‘fast-growing’ strains.

## Appendix A Supplementary data

Supplementary figures. Image 2

Supplementary Tables. Image 1
