# Supplementary material for: Proteomic comparison of selective breeding and growth hormone transgenesis in fish: Unique pathways to enhanced growth
Source: J Proteomics. 2019 Feb 10;192:114–24. doi: 10.1016/j.jprot.2018.08.013 (PMC7086150; doi:10.1016/j.jprot.2018.08.013)
Supplement: Supplementary file 2 — Supplementary Tables. [file mmc2.docx]

*Supplementary Information*

**Proteomic comparison of selective breeding and growth hormone transgenesis in fish: unique pathways to enhanced growth**


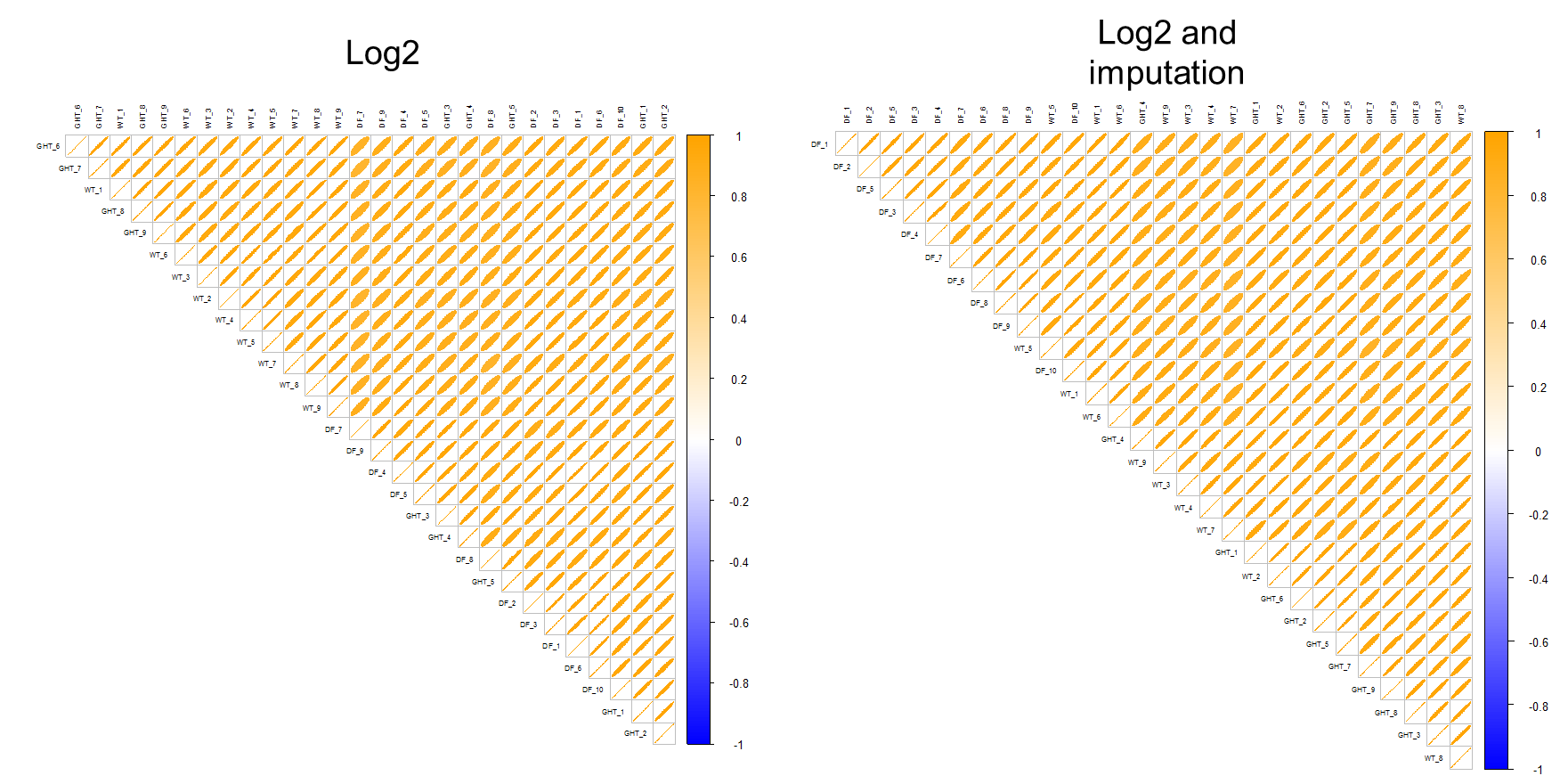


**Fig. S1**. Reproducibility of protein abundance estimates across samples (320 protein dataset used in statistical analysis), evidenced by Pearson correlation for all pairwise comparisons. Individual samples show reproducible abundance values for Log2-transformed LFQ values (data shown before and after imputation), evidenced by high correlation values and depicted by the small elliptical areas, which are inversely proportional to the R value.


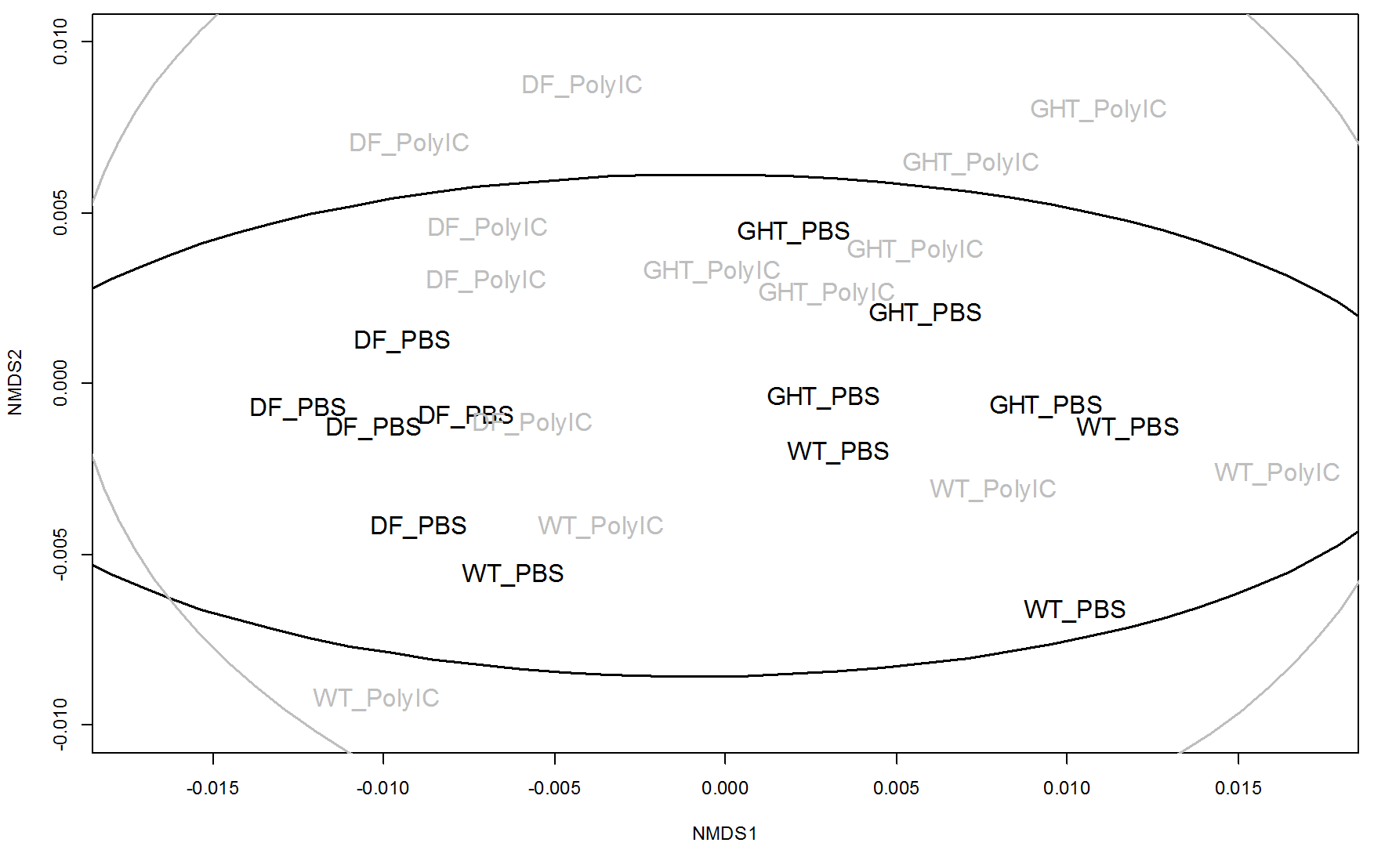


**Fig. S2**. Nonmetric multidimensional scaling (nMDS) showing lack of separation between fish injected with either Poly I:C or PBS. PBS-injected fish are coloured black; Poly I:C injected fish are grey. Each label represents one individual fish and their entire quantified muscle proteomic profile. Labels located nearer each other are more closely related in their proteomic profiles. Ellipses represent 95% confidence intervals.


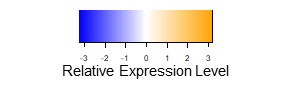

**Fig. S3**. Full heatmap of significantly differentially-expressed proteins contrasting GHT vs. WT. Each heatmap includes all tested fish individuals from the three different strains. Rows represent normalized Z-scores of log-2 transformed imputed LFQ values from MaxQuant. Accession numbers and protein names are from the O. kisutch and S. salar NCBI RefSeq databases, respectively.


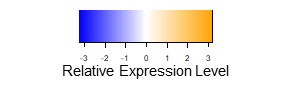

**Fig. S4**. Full heatmap of significantly differentially-expressed proteins contrasting DF vs. WT. Each heatmap includes all tested fish individuals from the three different strains. Rows represent normalized Z-scores of log-2 transformed imputed LFQ values from MaxQuant. Accession numbers and protein names are from the O. kisutch and S. salar NCBI RefSeq databases, respectively.


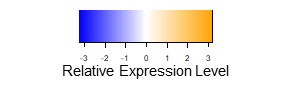

**Fig. S5**. Full heatmap of significantly differentially-expressed proteins contrasting DF vs. GHT. Each heatmap includes all tested fish individuals from the three different strains. Rows represent normalized Z-scores of log-2 transformed imputed LFQ values from MaxQuant. Accession numbers and protein names are from the O. kisutch and S. salar NCBI RefSeq databases, respectively.

**A**


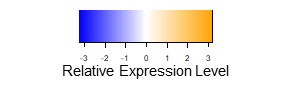


**
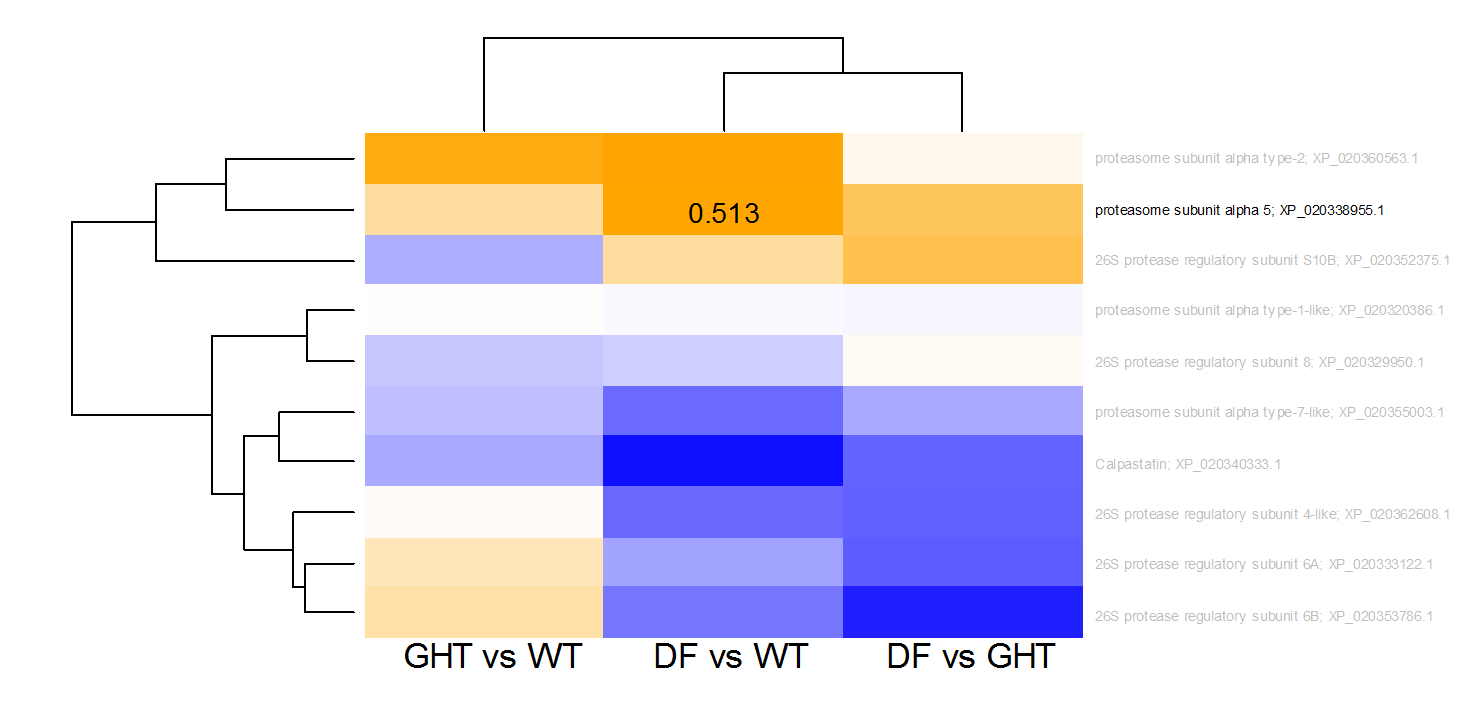
**

**B**


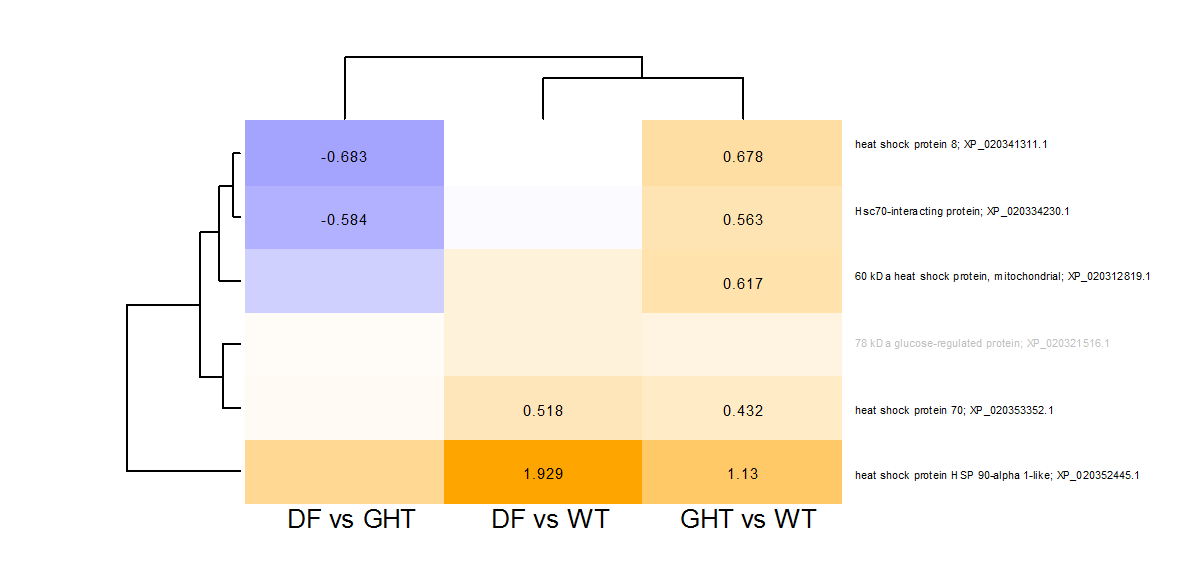


**Fig. S6**. Hierarchical clustering and heatmap analysis of: (**A**) proteins involved in protein catabolism and (**B**) molecular chaperones and heat shock protein family members. Protein titles in black show overall significant differential expression across the three strains. Log2 fold-changes in expression level are given only for proteins showing significant differential expression between the strain pairwise comparisons shown. Accession numbers and protein names are from O. kisutch and S. salar NCBI RefSeq databases, respectively.


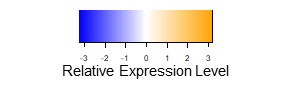


**Fig. S7**. Hierarchical clustering and heatmap analysis of proteins involved in sarcomeric organization and muscle contraction. Protein titles in black show overall significant differential expression across the three strains. Log2 fold-changes in expression level are given only for proteins showing significant differential expression between the strain pairwise comparisons shown. Accession numbers and protein names are from O. kisutch and S. salar NCBI RefSeq databases, respectively.
